# Graphene Oxide Based Magnetic Nanocomposites with Polymers as Effective Bisphenol–A Nanoadsorbents

**DOI:** 10.3390/ma12121987

**Published:** 2019-06-20

**Authors:** Kyriazis Rekos, Zoi-Christina Kampouraki, Charalampos Sarafidis, Victoria Samanidou, Eleni Deliyanni

**Affiliations:** 1 Laboratory of Chemical and Environmental Technology, Department of Chemistry, Aristotle University of Thessaloniki, 54124 Thessaloniki, Greece; rekoskyriazis@yahoo.gr (K.R.); zoickamp@chem.auth.gr (Z.-C.K.); 2 Laboratory of Physics, Department of Physics, Aristotle University of Thessaloniki, 54124 Thessaloniki, Greece; hsara@auth.gr; 3 Laboratory of Analytical Chemistry, Department of Chemistry, Aristotle University of Thessaloniki, 54124 Thessaloniki, Greece; samanidu@chem.auth.gr

**Keywords:** magnetic graphene oxide, polymers, bisphenol–A, adsorption

## Abstract

Magnetic graphene oxide was impregnated with polymers for the preparation of nanocomposite adsorbents to be examined for the adsorptive removal of a typical endocrine disruptor, bisphenol–A (BPA) from aqueous solutions. The polymers used were polystyrene, chitosan and polyaniline. The nanocomposites prepared were characterized for their structure, morphology and surface chemistry. The nanocomposites presented an increase adsorptive activity for BPA at ambient conditions, compared to pure magnetic oxide, attributed to the synergistic effect of the polymers and the magnetic graphene oxide. The increased adsorption of BPA exhibited by the nanocomposites with chitosan and polyaniline could be attributed to the contribution of amine groups.

## 1. Introduction

Endocrine-disrupting chemicals (EDCs) are chemical compounds that can endanger the human endocrine system, since they can imitate a natural hormone by catching their receptors or by interfering with the transport and metabolic processes of hormones in humans and animals [1,2,3]. Nowadays, the EDCs have revealed important global interests, due to the fact that a lot of types of EDC have been observed in water sources in the environment [4], such as drinking water, groundwater, surface water and wastewater [2,5].

Bisphenol–A (BPA), 2,2′-bis(4-hydroxyphenyl) propane, is an endocrine disruptor that for over 50 years was widely used in thermal paper production, as a monomer in the production of poly-carbonate plastics and epoxy resins as well as an additive in the formation of some polyvinyl chloride (PVC) plastics [6,7]. Among its properties are low vapor pressure, low volatility and moderate water solubility. Bisphenol–A can easily leach out of products and migrate into the aquatic environment [7], creating a public health, as well as environmental, threat worldwide [8]. 

Several methods, including adsorption [3,4], catalytic and biocatalytic oxidation [9,10], ozonation [11], photocatalysis [12] and membrane and biofilters processes [13,14], have been employed for removing BPA from waters. Adsorption is the most commonly used method due to its high efficiency, comparatively low cost and easy operation creating fewer harmful by-products [8]. Adsorbent materials include zeolites [15], mesoporous silica [16], montmorillonite hybrids [17,18] and several biowastes [4,19,20]. Besides, carbonaceous materials, like activated carbons, carbon nanotubes [21], graphene and graphene oxide [3] have proved to be remarkable adsorbates, as well. 

Recently, magnetic nanomaterials were investigated as adsorbents with the advantage of the magnetic solid-phase extraction (MSPE) method [22,23]. This method has considerable advantages in separation processes, due to the low cost and the satisfactory magnetic separation, in less time, avoiding filtration [24] and centrifugation procedures [25]. Iron-based materials are often used for environmental treatments applying magnetic solid-phase extraction (MSPE) with the most common to be magnetite (Fe_3_O_4_) because of its low cost, easy separation, high surface area and the fact that it is friendly with the environment [26,27,28]. Magnetic oxides like Fe_3_O_4_, CoFe_2_O_4_ and NiFe_2_O_4_ can be also used as a magnetic source [24]. 

In the current study, the advantage of magnetic solid-phase extraction (MSPE) was applied to a carbonaceous material, graphene oxide, that was impregnated with polymers in order for its surface chemistry as well as its texture to be improved. The examined polymers were polystyrene, polyaniline and chitosan. The nanocomposites prepared were examined for bisphenol–A adsorption from aqueous solutions. Several parameters affecting adsorption were examined, i.e., the effect of pH, the effect of ionic strength, the effect of contact time and the effect of BPA initial concentration. The characterization of the nanocomposites was carried out by x-ray diffraction (XRD), N_2_ adsorption (BET), scanning electron microscopy (SEM) and Fourier infrared spectroscopy (FTIR). These measurements contributed to the understanding of the adsorption mechanism and the beneficial effect of the magnetite and the polymers on BPA adsorption. 

## 2. Materials and Methods 

### 2.1. Chemical Reagents

Graphite powder, sulfuric acid (H_2_SO_4_, 98% wt.), potassium permanganate (KMnO_4_), hydrogen peroxide (H_2_O_2_ 30% *w*/*w*), hydrochloric acid (HCl, 37% ar.), ferric chloride (FeCl_3_·6H_2_O), ferrous chloride (FeCl_2_·4H_2_O), ethanol (CH_3_CH_2_OH, absolute), acetic acid (CH_3_COOH, glacial), N′-N′-dimethylformamide (C_3_H_7_NO, DMF), polystyrene, aniline (C_6_H_5_NH_2_), ammonium persulfate ((NH_4_)_2_S_2_O_8_) and bisphenol–A ((CH_3_)_2_C(C_6_H_4_OH)_2_) were purchased from Sigma Aldrich (St. Louis, MO, USA). Ammonium solution (25% as NH_3_) and potassium hydroxide (KOH) was supplied by Panreac AppliChem (Darmstadt, Germany). Glutaraldehyde (GLA) (C_5_H_8_O_2_, 50 wt.% in water) was obtained from J&K Scientific GmbH (Germany). Acetonitrile (CH_3_CN) HPLC gradient grade was supplied by Sigma Aldrich. Chitosan was purchased from Sigma Aldrich and had high molecular weight with >75% deacetylation. All the reagents were analytical grade (puriss. pa. ≥ 98.5%) and were used without further purification.

### 2.2. Materials Preparation

#### 2.2.1. Graphite Oxide (GO) Preparation

Graphite oxide (GO) was synthesized according to a modified Hummers method [29]. An amount of graphite (10 g) and 230 mL of 98% (*w*) H_2_SO_4_ were mixed under stirring (30 min at 0 °C) and then 30 g of KMnO_4_ were added into the suspension, keeping the dispersion below 20 °C, until it gradually turned to a brownish slurry. After that point, 230 mL of water were initially added and at a second stage, 1400 mL of water and 100 mL of H_2_O_2_ (30% (*w*)). After centrifuging, the solid produced was washed with HCl (10% (v)), deionized water and was freeze-dried. GO was obtained as a dark brown powder.

#### 2.2.2. Preparation of Magnetite Nanoparticles (Fe_3_O_4_)

For the synthesis of magnetite nanoparticles [6,30], FeCl_2_·4H_2_O and FeCl_3_·6H_2_O were added at a mass ratio 0.35:1 into a flask with 100 mL of deionized water, at 60 °C in inert atmosphere under stirring at 400 rpm for 1 h. An aqueous ammonium solution was dropwise added under vigorous stirring until the pH of the solution was measured to be 10. The suspension was heated and further kept at 90 °C under stirring for 1 h; after filtration, washing with distilled water until neutral pH followed by ethanol washing it was freeze-dried. 

#### 2.2.3. Synthesis of Graphite Oxide/Cross-Linked Chitosan (GO–CS) and Graphite Oxide/Cross-Linked Chitosan/Magnetite (GO–CSm) Composites

For the preparation of the GO–cross-linked chitosan (CS) composite [31], chitosan solution (2% *w*/*v*) was prepared by dissolving a specific amount of powder chitosan (2 g) into acetic acid solution (100 ml 2% *w*/*v*) under ultrasonic stirring for 2 h. For the cross-link of the chitosan in order to avoid swelling, GLA (50 wt.% in water) was added into the suspension (3 mL GLA per 0.4 g of CS). GO was then added at a 75% GO:CS ratio, and the mixed system was under stirring for 90 min at 50 °C in a water bath. The pH of the reaction system was adjusted to 9–10 with micro-additions of NaOH (0.1 mol/L) and kept in the water bath for further 1 h at 80 °C. The black solid was produced after ethanol and distilled water washing in turn until the pH became neutral and was dried in a vacuum oven at 50 °C. The final product was graphite oxide/chitosan (GO–CS) nanocomposite. 

For the preparation of GO–cross-linked chitosan/magnetite (CSm) composite, an amount of chitosan (2 g) was dissolved in 100 mL of acetic acid (2% *v*/*v*) and sonicated for 30 min [32]. An amount of magnetite (0.75 g) was added, and after 2 h stirring, 1.5 g of GO was further added to the reaction flask followed by the addition of GLA (15 mL, 50 wt % in water) and pH adjustment to 9–10 with a dilute NaOH aqueous solution (0.1 mol/L). After further stirring for 1 h at 80 °C, the precipitate washed with ethanol and distilled water and dried at 50 °C. The prepared nanocomposites (GO–CS and GO–CSm) were ground to 75–125 μm particle size.

#### 2.2.4. Synthesis of Graphite Oxide/Polystyrene (GO-PS) and Graphite Oxide/Polystyrene/Magnetite (GO-PSm) Composites

For the preparation of graphite oxide/polystyrene composite, polystyrene was dissolved in N, N-dimethylformamide at 60 °C under stirring for 30 min. Then, GO was added to the solution at a 75% GO:PS ratio. The mixture kept under vigorous stirring for 60 min and then was ultrasonicated for further 30 min. We added 200 mL of an 1:10 DMF aqueous solution, for the agglomeration and flocculation of the material. The product was filtrated, washed with deionized water and freeze-dried. 

For the magnetic composite (GO–polystyrene/magnetite (PSm)) preparation, the previously described process was followed. An amount of magnetite was added (0.375:1 Fe_3_O_4_:PS mass ratio) prior to the sonication step. The prepared nanocomposites (GO–PS and GO–PSm) were ground to fine powders of 75–125 μm particle size.

#### 2.2.5. Synthesis of Graphite Oxide/Polyaniline (GO–PANI) and Graphite Oxide/Polyaniline/Magnetite (GO–PANIm) Composites

For the graphite oxide/polyaniline (GO–PANI) synthesis, GO (500 mg) was dispersed in 25 mL DI water and after 10 mins ultrasonication, 7 mmol of aniline was added [33]. The mixture was further stirred at 25 °C for 10 min and in an ice bath (0–5 °C) for 30 min. Then, 7 mmol of ammonium persulfate aqueous solution was added and the polymerization reaction proceeded for 12 h at a temperature between 0–5 °C. The GO–PANI composite produced was freeze-dried after washing.

For the magnetic composite preparation, the previously described process was followed. Additionally, an amount of magnetite was added (0.375:1 Fe_3_O_4_:PANI mass ratio) before the step of sonication. The prepared nanocomposites (GO–PANI and GO–PANIm) were ground to fine powders of 75–125 μm particle size.

### 2.3. Characterization Techniques—Instrumentation

X-ray diffraction (XRD, Rigaku-Miniiflex II, Neu-Isenburg, Germany) patterns were obtained using powder diffraction techniques on a Philips PW1820 diffractometer with CuK(alpha) radiation, to observe the composition, structure and physical properties of the materials. The samples were scanned from 5° to 70°. The surface structure and morphology of the samples were obtained by using scanning electron microscope (SEM, Zeiss Supra 55VP, Jena, Germany), at an accelerating voltage 15.00 kV. The magnetic properties of the composites were analyzed using a PAR Model 151 (Princeton Applied Research Corporation, Princeton, NJ) vibrating sample magnetometer (VSM) calibrated against a NIST-certified Ni standard at room temperature. Inductively coupled plasma mass spectrometry (ICP-MS, Agilent 7500s-Agilent Technologies, Waldbronn, Germany) measurements were carried out in order to detect iron concentration and thus magnetite concentration in the nanocomposites prepared. ^57^Fe Mössbauer spectra were recorded at RT in a conventional transmission geometry apparatus operated in triangular wave mode with a ^57^Co in Rhodium matrix source and calibrated with an α-Fe reference sample. In addition to the above methods, there are also highly sensitive quartz methods for determining the magnetic properties of materials as described in the references [34,35]. 

Surface chemistry was examined by Fourier transform infrared (model FTIR-2000, Perkin Elmer, Dresden, Germany). A PerkinElmer-2000 FTIR spectrometer with the KBr disks method was the instrument used with the spectra to be recorded from 4000 to 400 cm^−1^ at the transmittance mode.

Specific Surface Area and pore structure of the graphene oxide—polymer materials were determined by the nitrogen adsorption–desorption isotherms at liquid N_2_ temperature (77 K), measured by the Quantachrome Autosorb-1C instrument was used with the samples to be overnight degassed at 25 °C and 10^−4^ Torr. 

A Mettler Toledo T50 titrator was used for potentiometric titration measurements. The nanocomposite sample (0.1 g in 50 mL of NaNO_3_ solution 0.01 mol/L), after 24 h stirring was titrated with 0.1 mol/L NaOH in inert atmosphere. The surface charge, Q (mmol/L), was estimated by the equation (Equation (1)) [36]:

(1) $$Q=\frac{C_{A}+C_{B}+\left[OH^{-}\right]+\left[H^{+}\right]}{W},$$

with *C_A_* and *C_B_* the acid and base concentrations (mol/L), respectively, [H^+^] and [OH^−^] the equilibrium concentrations of these ions (mol/L), and W the solid concentration (g/L).

### 2.4. Adsorption Experiments

For the investigation of the prepared materials’ adsorption capacity, batch BPA adsorption experiments were run in triplicate. The studied parameters were: initial pH, ionic strength (in form of solid NaCl addition), initial BPA concentration, contact time and desorption efficiency. 

#### 2.4.1. Effect of pH and Ionic Strength on BPA Adsorption

The experiments for the influence of the initial pH were performed by dispersing 0.01 g of the under examination nanocomposite in 20 mL (V) of BPA solution (C_o_ = 100 mg/L) in conical flasks. The pH was ranged from 3 to 9 and was constant through the adsorption process. The flasks were shaken into a water bath for 24 h at 25 °C. 

The effect of ionic strength was tested by dispersed 0.01 g of the relative nanocomposite in 20 mL (V) of BPA solution (C_o_ = 100 mg/L) in in conical flasks. The salt concentrations examined were between 0.01 and 1 M. The flasks were shaken into a water bath for 24 h at 25 °C. 

The optimum pH value and ionic strength was testified by the amount of the remaining BPA concentrations. The suspensions of the non-magnetic nanocomposites, after adsorption, were centrifuged (at 7500 rpm for 10 min) and the supernatant solution was filtered through a 0.45 μm cellulose membrane (purchased by Schleicher-Schuell MicroScience) while the magnetic solid-phase extraction was applied for the magnetic nanocomposites. High-performance liquid chromatography (HPLC) with UV detection at 230 nm was applied to measure the remaining concentration of BPA in the solution. A C_18_ column (PerfectSil Target ODS-3, 250 × 4.6 mm 5 μm, purchased by MZ-Analysentechnik GmbH, Mainz, Germany) was used as stationary phase operating at ambient temperature. Isocratic elution was applied using a mobile phase of a water and acetonitrile mixture (70% CH_3_CN/30% H_2_O), at a flow rate of 1.0 mL/min. The back-pressure observed was not higher than 100 bar. Retention time was used to identify the presence of Bisphenol A in the solution, compared with that of the reference standard solution under the same chromatographic conditions. The HPLC instrumentation was consisted of an LC-10AD pump (Shimadzu, Kyoto, Japan), a Rheodyne 7125 injection valve (Rheodyne, Cotati CA, USA) with a 20 μL loop, and an SSI 500 UV-vis detector (SSI, State College, PA, USA), which was operating at a sensitivity setting of 0.002 AUFS.

The removal percentage (R%) of BPA was calculated according to the Equation (2):

(2) $$R\%=\frac{C_{o}-C_{e}}{C_{o}}*100,$$

where C_o_ and C_e_ (mg/L) are initial and equilibrium concentrations of BPA, respectively [37]. 

#### 2.4.2. Effect of Contact Time

The experiments for the influence of the contact time were performed by dispersing 0.01 g of the under examination nanocomposite in 20 mL (V) of BPA solution (C_o_ = 100 mg/L) in conical flasks. The pH was fixed to three and was constant through the adsorption process. The flasks were shaken into a water bath at 25 °C for different time duration. The pseudo-first (Equation (3)) and the pseudo-second order (Equation (4)) kinetic models were applied to the experimental results.

The linearized-integral form of the pseudo first-order model is given by the following equation [38]:

(3) $$q\left(t\right)=q_{e}\left[1-\exp\left(-k_{1}t\right)\right],$$

where *q_e_* and *q_t_* are the amounts of BPA adsorbed at equilibrium and at time *t* (mg/g), respectively, and *k*_1_ is the Lagergren rate constant of adsorption (min^−1^). 

The form of the pseudo-second order model is

(4) $$q\left(t\right)=q_{e}\frac{k_{2}q_{e}t}{1+k_{2}q_{e}t},$$

where *k*_2_ is the pseudo-second order rate constant of adsorption (g/mg·min) [39,40]. 

#### 2.4.3. Desorption Experiments

Desorption was also studied in order the ability of the nanocomposites for reuse to be examined. The experiments performed with BPA-loaded sorbents, dispersing 0.01 g with 20 mL of deionized water for a pH range 3–9, at 25 °C for 24 h. The scope of the experiments was the determination of the better desorption pH value of the graphene-oxide-based materials. Besides, organic solvents (acetonitrile and methanol) were also tested as eluents. 

## 3. Results

### 3.1. Sorbent Characterization

#### 3.1.1. XRD Measurements

**Figure 1:**
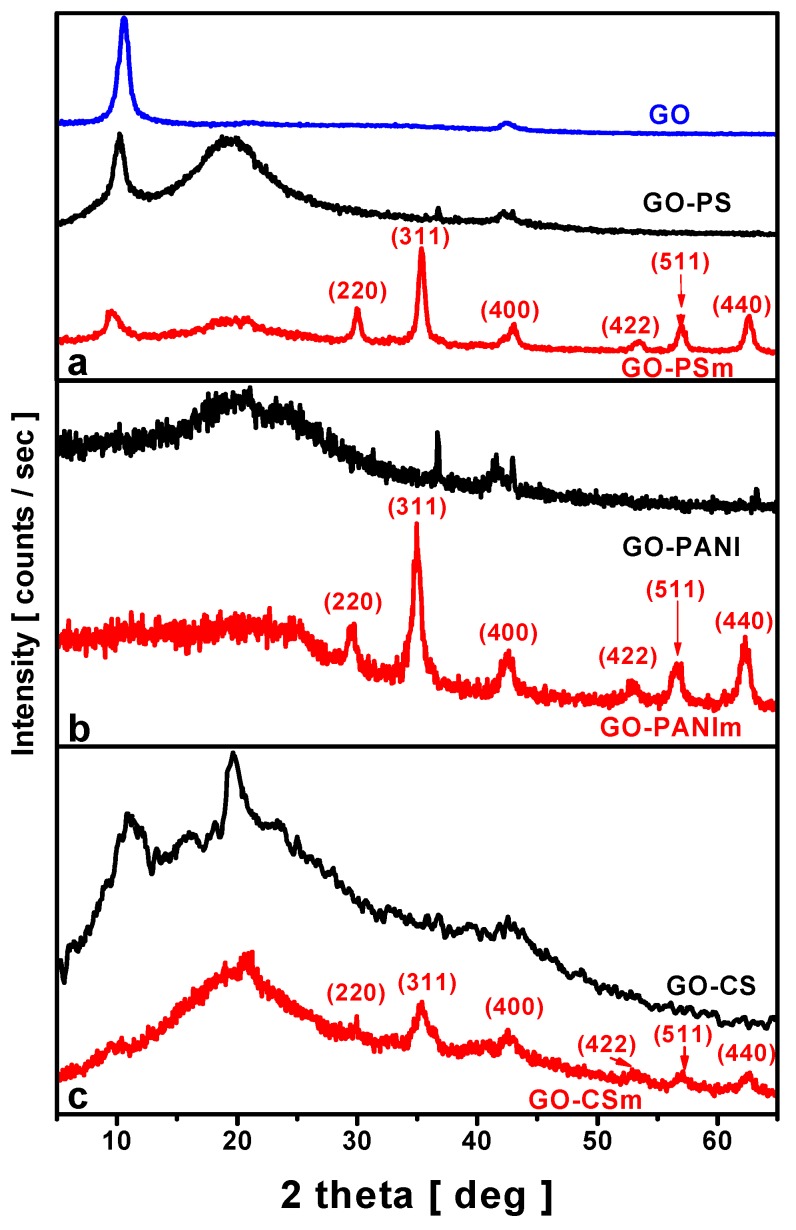
X-ray diffraction pattern of composed materials from 5° to 65°. (**a**) GO-PS and GO-PSm; (**b**) GO-PANI and GO-PANIm; (**c**) GO-CS and GO-CSm.

X-ray diffraction patterns of the composites were measured from 5° to 60° 2*θ* and are shown in Figure 1. For the magnetic nanocomposites, the characteristic diffraction peaks of magnetite at 2*θ* (degree) of 29.9°, 35.3°, 43.2°, 53.5°, 57.1° and 62.7° (JCPDS 19-0629) indicate the successful preparation of magnetite impregnated to the materials prepared [41]. 

The XRD patterns for GO-PS and GO–PSm nanocomposites (Figure 1a) showed the characteristic peak of polystyrene at 2*θ* = 19.8° [42], while for the GO-PANI and GO–PANIm nanocomposites the broad peaks centered at 2*θ* = 13°, 20.6° and 25.1° (Figure 1b) corresponded to the polyaniline crystal planes [43]. The XRD patterns of GO–CS and GO–CSm nanocomposites (Figure 1c) revealed a broad peak at 2*θ* = 20.2° as a result of the amorphous phase of chitosan, that was not altered after the addition of GO [31].

In all patterns, the characteristic peak of GO at 2*θ* = 9.98°, was eliminated or disappeared proving that the successful impregnation of the polymers into the graphite oxide layers led to GO exfoliation.

#### 3.1.2. Surface Properties—Morphology

The porosity of an adsorbent offers information for its textural parameters and the available area for adsorption and nitrogen adsorption measurements are used for the characterization of the porosity. The values for the specific surface area and pore volume revealed by the under-examination adsorbents were calculated by nitrogen adsorption measurements and are presented in Table 1. N_2_ adsorption–desorption isotherms for GO showed a surface area (SSA) of 20.93 m^2^/g [3]. The impregnation of GO with the polymers resulted to the reduction of the total pore volume for the polystyrene and chitosan nanocomposites, but to a very significant increase for the polyaniline nanocomposites. For all materials the micropore volume was eliminated. The reduction of the pore volume may be due to the reactions of the polymers’ amino groups with the surface groups of GO and the development of new products inside their pores [30]. For all the magnetic nanocomposites, an increase of specific surface area was noticed possibly attributed to the impregnation of magnetite that may contributed with its surface area. 

**Figure 2:**
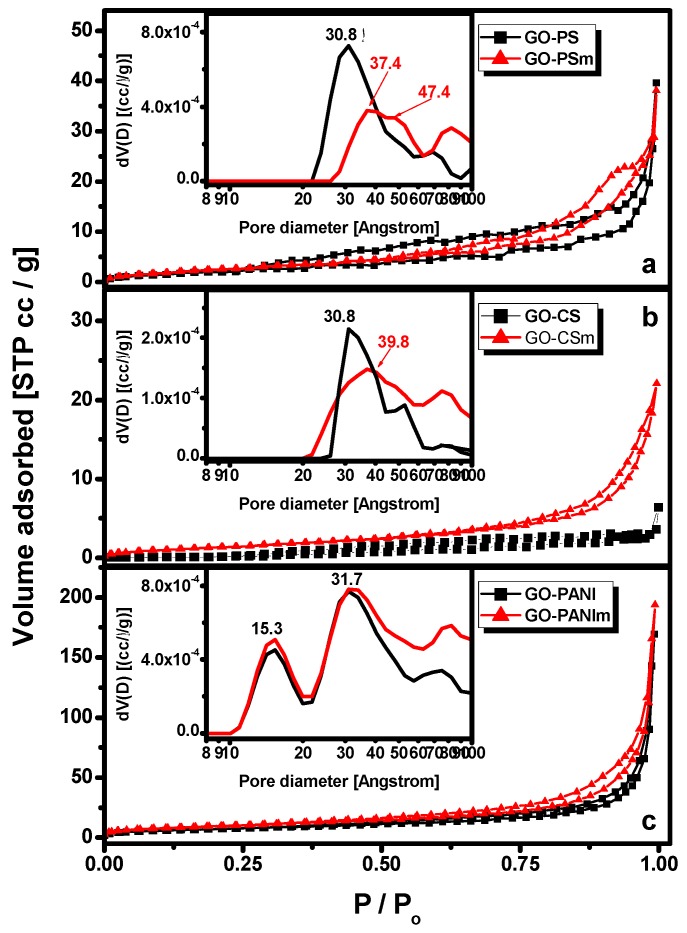
N_2_ adsorption–desorption isotherms and pore size distribution (inset figures). (**a**) GO-PS and GO-PSm; (**b**) GO-CS and GO-CSm; (**c**) GO-PANI and GO-PANIm.

The pore size distributions for the GO-polymers nanocomposites were calculated by the Density Functional Theory (DFT) and are shown in the relative insets of Figure 2a–c. As it appears in the Figures, the mean diameter of the main pores, for all nanocomposites, was at the mesoporous range (about 3.5 nm), according to IUPAC classification [44]. Since the average length of the BPA molecule is 0.94 nm [45] the pore size of all composites was sufficient for the adsorptive molecules to reach the composites pore system.

**Figure 3:**
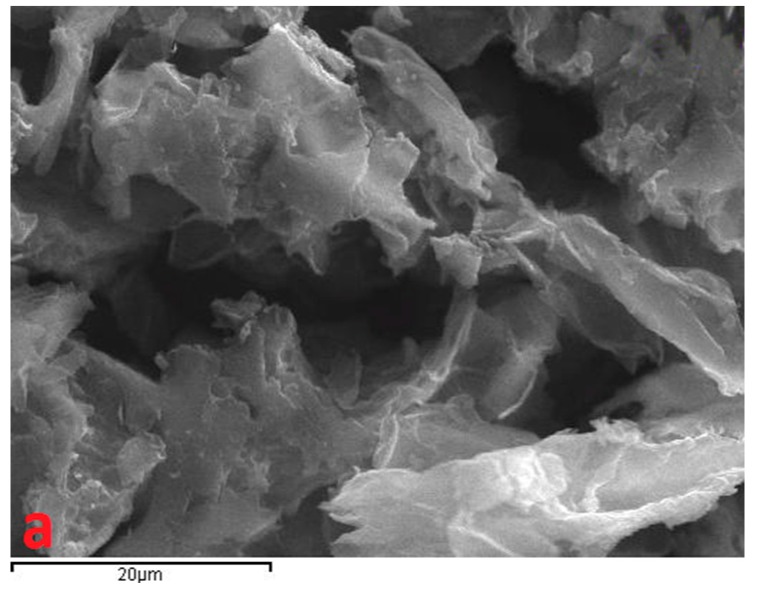

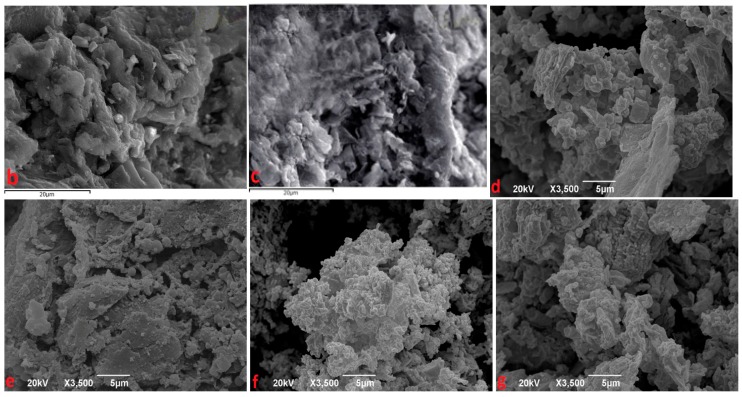
Scanning electron microscope (SEM) images of graphite oxide (GO) (**a**), GO–cross-linked chitosan (CS) (**b**), GO–cross-linked chitosan/magnetite (CSm) (**c**), GO–polystyrene (PS) (**d**), GO– polystyrene/magnetite (PSm) (**e**), GO–polyaniline (PANI) (**f**), GO–polyaniline/magnetite (PANIm) (**g**).

The SEM micrographs, presented in Figure 3, showed that GO displayed typical rippled and crumpled surfaces indicative of graphene. After the polymer impregnation, the nanocomposites were divided into fragments. On the other hand, spheres were observed in the micrographs of the magnetic nanocomposites that were possibly linked to the magnetite.

#### 3.1.3. Magnetization Measurements (VSM and ^57^Fe Mössbauer)

**Figure 4:**
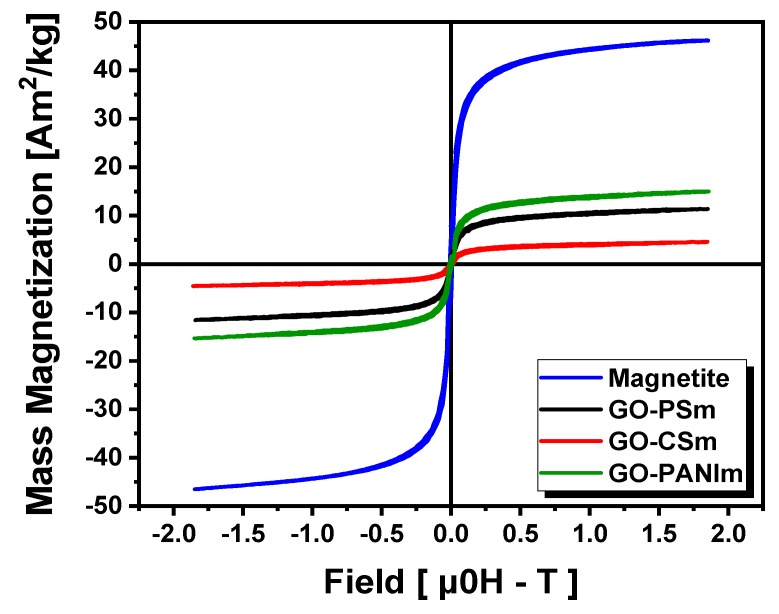
Magnetization curves for the magnetic composites; a pure magnetite loop is included for reference.

The magnetic properties of the composites are crucial for their role in the magnetic separation process. The incorporation of the magnetic nanoparticles and the stability of the composite directly affects the magnetic energy of the material within an external magnetic field and this is reflected in saturation magnetization (Ms). Higher Ms values may enhance the efficiency in magnetic separation. The magnetic properties of the three magnetic GO-polymer nanocomposites (GO–PSm, GO–CSm and GO–PANIm) were investigated by recording the field dependent magnetization hysteresis curves at room temperature and in external fields up to 2 T. As it can be seen from Figure 4, it is evident from the shape of the loops that all magnetic nanocomposites possess superparamagnetic characteristics at room temperature [30]. Besides, they presented similar hysteresis loops (i.e., initial slope, curvature, saturation plateau) with the only difference being the position of the saturation plateau that indicate the magnetite content of the nanocomposites. The VSM results of magnetite content were consistent with the ICP–MS measurements from which the iron presence was investigated and the results were 11.74%, 5.96% and 15.64% *w*/*w* (g Fe/100 g sample) for GO–PSm, GO–CSm and GO–PANIm samples, respectively. Ms values were macroscopic and characterize the entire sample. However, a correlation with Fe presence as determined by ICP–MS measurements is expected. Indeed, with reference to the GO–CSm sample, the PANIm:PSm:CSm ratio was 2.7:2.2:1.0 for the Ms values and 2.6:2.0:1.0 for the Fe presence; these results were in excellent agreement within experimental uncertainty. 

**Figure 5:**
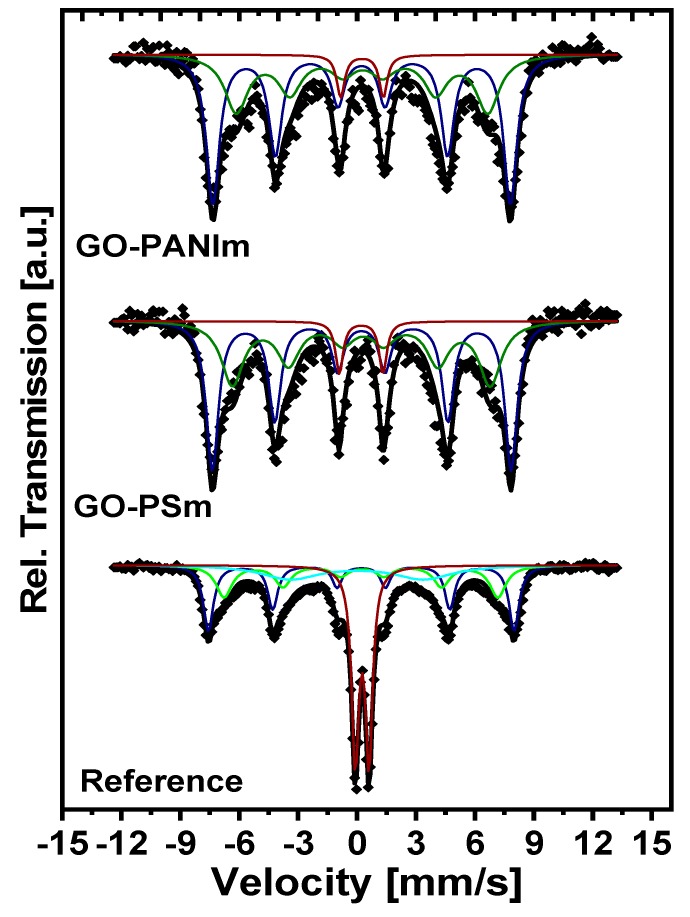
^57^Fe Mössbauer spectra of GO–PANIm, GO–PSm and magnetite as reference.

^57^Fe Mössbauer spectra were collected at RT and they are presented in Figure 5. All samples were fitted with a composite model which included two sextets and a doublet. For the case of the GO–CSm sample the signal was too weak resulting in a noisy spectrum; thus, this sample is not included here. As seen in Figure 5, the original reference sample presented a large doublet accounting for the 25% of the total integrated intensity. This doublet can be attributed in superparamagnetic relaxation effects due to the particles’ size. GO–PANIm and GO–PSm samples present very similar spectra. The best fit was obtained with two broad sextets and a doublet. The sextets had almost identical parameters, hyperfine fields (BHF) were in the range of 47.0–47.2 T for the first sextet and 39.7–40.8 T for the second sextet, which confirmed the stability of the Fe oxide in both composites. The smaller values of BHF for the GO–PANIm and GO–PSm samples compared to the Reference sample can be attributed to the overall weakening in magnetic interactions which was also reflected in the saturation magnetization, probably due to the bonding between the Fe and the C atoms. Isomer shift (δ) values were 0.23 mm/s for the first sextet in both samples and 0.26–0.27 mm/s for the second sextet, all isomer shift values are relative to α-Fe. Quadruple splitting (Δ) values were very small as expected due to the cubic symmetry of magnetite.

### 3.2. Adsorption Results

The nanocomposites were examined for their ability for BPA adsorption; the effect of parameters like pH, ionic strength, initial BPA concentration and contact time was examined.

#### 3.2.1. Effect of pH

**Figure 6:**
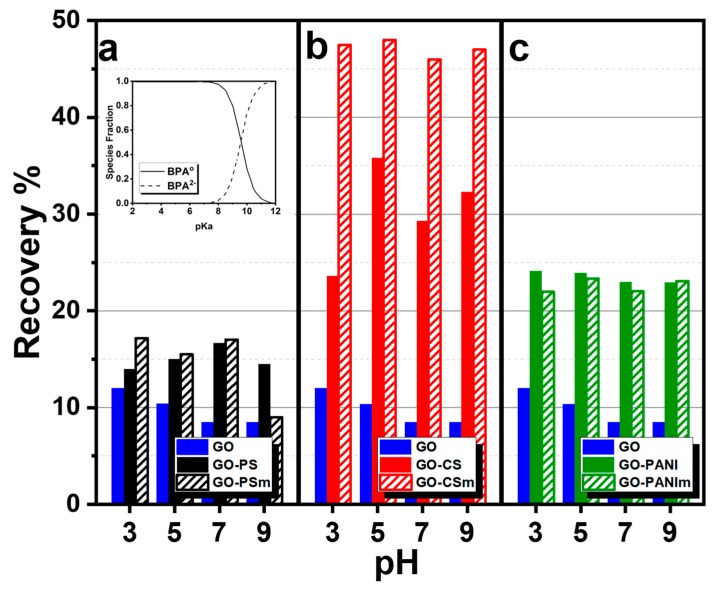
Effect of pH on adsorption of bisphenol–A (BPA) by (**a**) GO–PS and GO–PSm, (**b**) GO–CS and GO–CSm and (**c**) GO–PANI and GO–PANIm, and GO as reference; in the inset: the species distribution diagram for BPA [46,47].

The pH of a solution affects the surface charge as well as the dissociation state of an adsorbed substance. Figure 6a presents the influence of pH on BPA removal for pH range from 3 to 9, on GO–PS and GO–PSm, Figure 6b on GO–CS and GO–CSm and Figure 6c on GO–PANI and GO–PANIm. 

**Figure 7:**
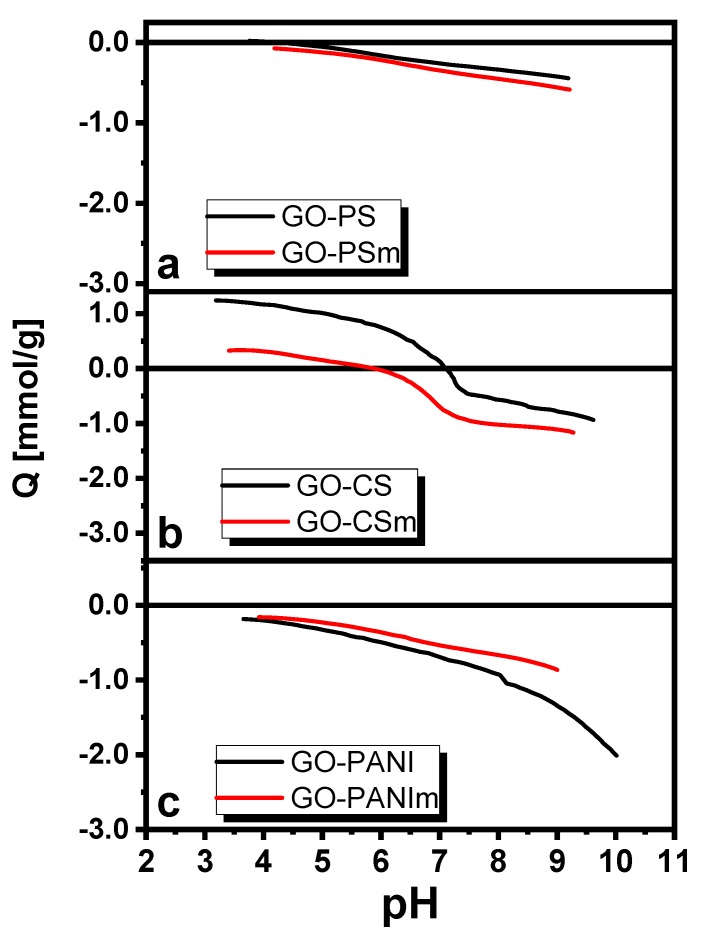
Potentiometric titration results for (**a**) GO–PS and GO–PSm, (**b**) GO–CS and GO–CSm, (**c**) GO–PANI and GO–PANIm.

The influence of pH for BPA removal on GO for pH range 3–9 is also presented in Figure 6 for the sake of comparison. With the pH increase from 3 to 9, the adsorption capacity of GO decreased, with the maximum uptake to be achieved at pH 3.0 and the percentage decrease of the removal efficiency from 37% at pH to 26% at pH 9.0. For the under examination nanocomposites, a slight adsorption capacity effect was observed at this pH range. This could be attributed to the fact that bisphenol A, with a value of pKa = 10.6 [47], as presented in the species distribution diagram (Figure 6 inset) [46,47], in aqueous solutions and at pH values < 10.6, exists in its molecular form while for pH values >10.6 it is present as HBPA^−^ or as BPA^2−^. The reduction of adsorption attributed to electrostatic repulsion forces could be created when pH > pKa = 10.6. From the potentiometric titrations results, presented in Figure 7, it is seen that the nanocomposites’ surface was positively charged for the whole pH range, except these with chitosan. For this reason, for the pH range tested, adsorption could be attributed to π–π and hydrophobic interactions. Since the maximum removal for GO occurred at pH 3, equilibrium and kinetic experiments were performed at this pH. 

#### 3.2.2. Effect of Ionic Strength

**Figure 8:**
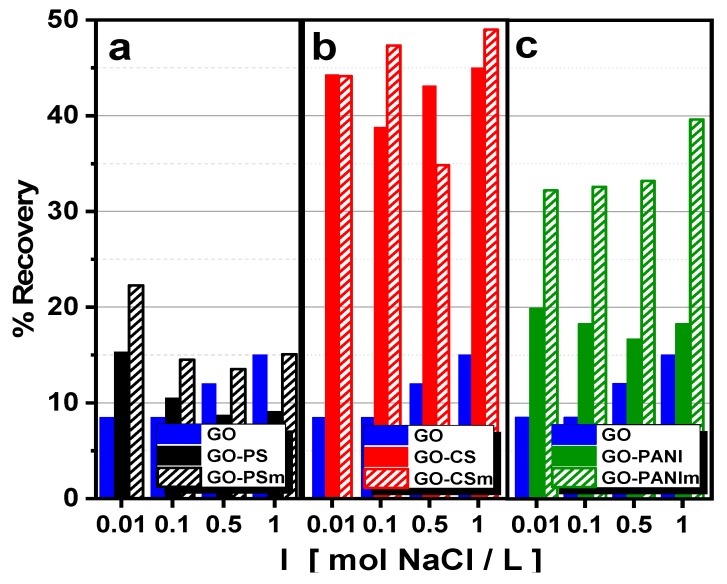
Effect of ionic strength on adsorption of BPA by (**a**) GO–PS and GO–PSm, (**b**) GO–CS and GO–CSm and (**c**) GO–PANI and GO–PANIm, and GO as reference.

Wastewaters have significant concentrations of salts, thus, the influence of the ionic strength on the BPA adsorption by the materials prepared was also examined. The adsorption capacity of the nanocomposites was slightly positively affected by the NaCl concentration, as presented at Figure 8, may be due to a “salting-out effect” that leads to a BPA solubility decrease, leading to an increase of BPA adsorption [48]. The NaCl concentration of ionic strength for all experiments was I = 1 mol/L, since from the experimental results, presented at Figure 8, it was obvious that the adsorption capacity of all nanocomposites was improved at this concentration value.

#### 3.2.3. Effect of Contact Time

**Figure 9:**
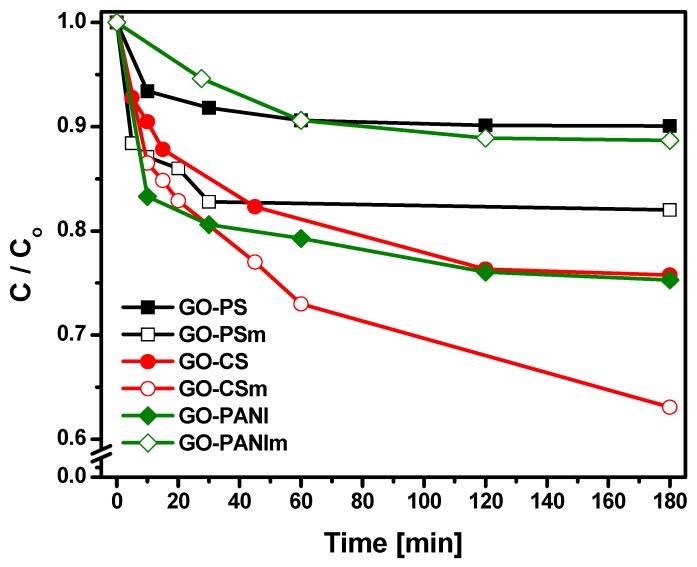
Influence of contact time on the adsorption of BPA.

**Figure 10:**
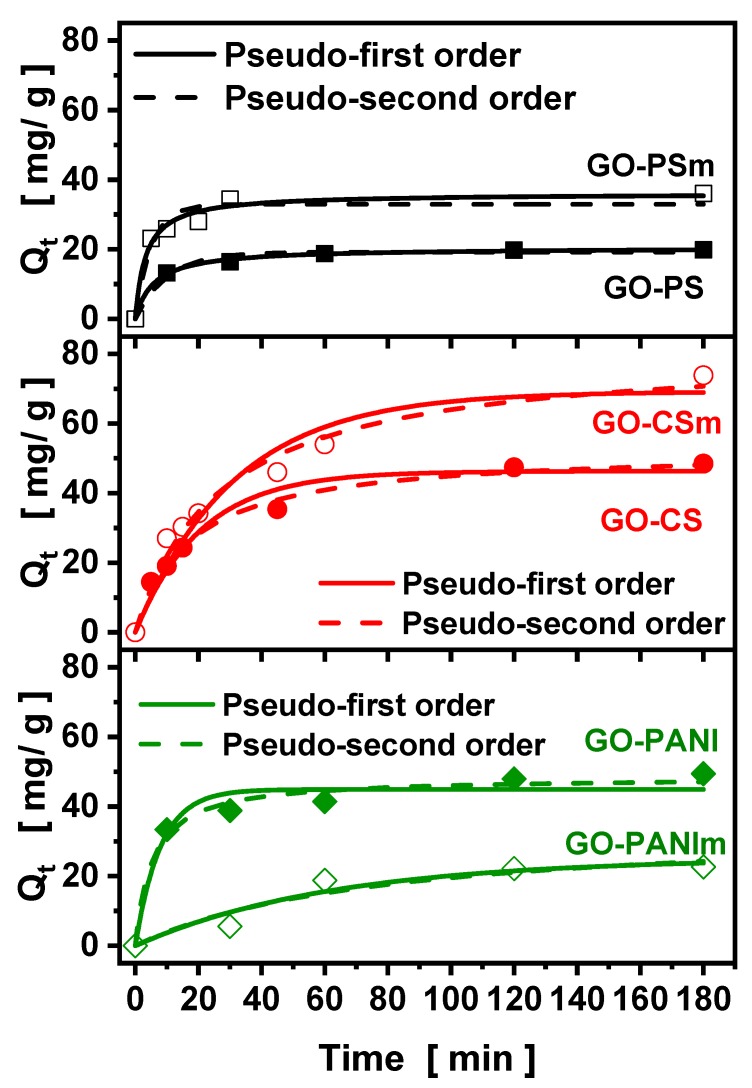
Pseudo-first and pseudo-second order kinetic model fitting.

The influence of contact time on BPA adsorption capacity of the nanocomposites prepared is presented at Figure 9. The equilibrium point for all materials was achieved at about 1 h, indicating that all materials showed a quick adsorption. The results were fitted to the pseudo-first and pseudo-second-order kinetic models (Figure 10). In Table 2 the parameters of the pseudo—first and pseudo—second order kinetic models are presented. The correlation coefficient values (R^2^) for the pseudo-second-order model were higher than those for the pseudo-first-order model, indicating that the pseudo-second-order kinetic model stimulated a fitting for the adsorption of BPA on the nanocomposites [39,40].

#### 3.2.4. Effect of Initial Concentration

**Figure 11:**
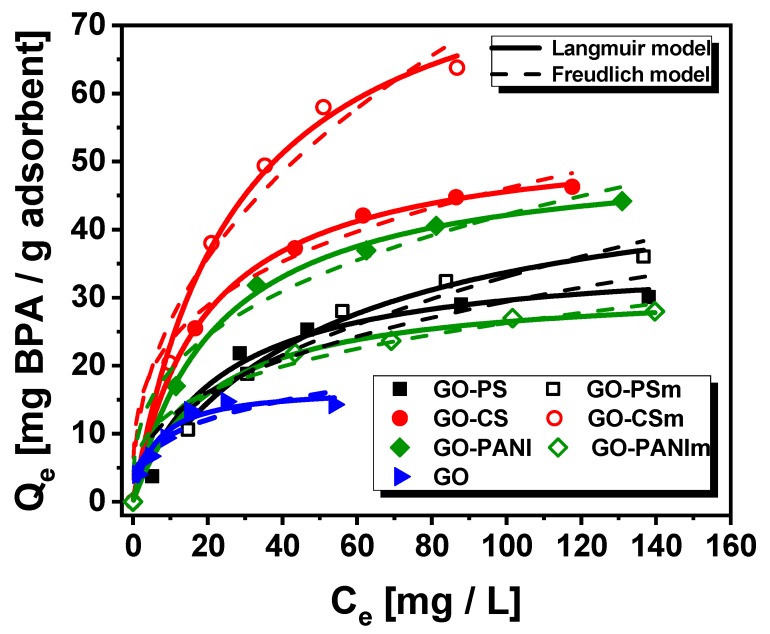
Isotherms of adsorption of BPA onto GO composites (compact lines for Freundlich equation fitting, dashed lines for Langmuir equation fitting.).

Experiments addressing the effect of the BPA initial concentration were performed for all nanocomposites as well as for GO (for comparison reason) and the results are shown in Figure 11. The results were fitted to Freundlich and Langmuir models and the relative parameters are presented in Table 3. The results in Figure 11 indicate that the nanocomposites at pH 3 presented as adsorbents, an increase in BPA removal compared to GO, with the GO–CSm to present the highest one that was 86.22 mg BPA per gram of the solid adsorbent, while the order of the removal was GO < GO–PANIm < GO-PS < GO–PSm < GO-PANI < GO-CS < GO–CSm. For all nanocomposites, the maximum removal they achieved was higher than that of GO indicating the beneficial contribution of the polymers’ impregnation.

Besides, a comparison was made for the values of the maximum adsorption capacities obtained from the Langmuir model of the nanocomposites studied with relative values reported in the literature. As presented in Table 4, the Q_max_ of the better performed nanocomposite GO–CSm (86.22 mg/g) was sufficiently higher. Considering the advantage of the magnetic separation from the mixture solution, the nanocomposites prepared can be successfully used in treatment processes for BPA removal.

#### 3.2.5. Desorption

**Figure 12:**
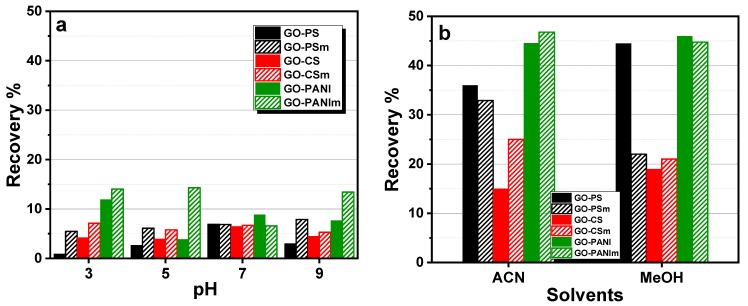
Effect of pH (**a**) and organic solvents (**b**) on BPA elution from GO-polymer composites.

The elution ability of the loaded nanocomposites was tested in order to assess their environmental safety. Deionized water, at a range of pH values, as well as methanol and acetonitrile were used as eluents for BPA elution from the loaded nanocomposites. The results are presented in Figure 12. The nanocomposites presented a negligible desorption capacity for the whole pH range. The organic solvents that were also tested for BPA elution from the adsorbents, were found to be more effective, with methanol being the optimal eluent.

### 3.3. FTIR Measurements

For the interpretation of the adsorption results of the materials, the surface chemistry characteristics of the materials before and after BPA adsorption were studied. 

The FTIR spectrum for GO has been previously reported [3,55,56,57]. Briefly, the bands at ~1050 and ~1730 cm^−1^ corresponded to the stretch vibration of C–O from C=O (carbonyl) and O–C=O (carboxyl), respectively, a band at 1240 cm^−1^ to C–O vibrations in epoxides and a band at ~1350 to sulfones or sulfates (S=O asymmetric stretching). The band at approximately 1600 cm^−1^ was indicative of the sp^2^ carbon skeletal network (aromatic rings) [3,55,56,57].

**Figure 13:**
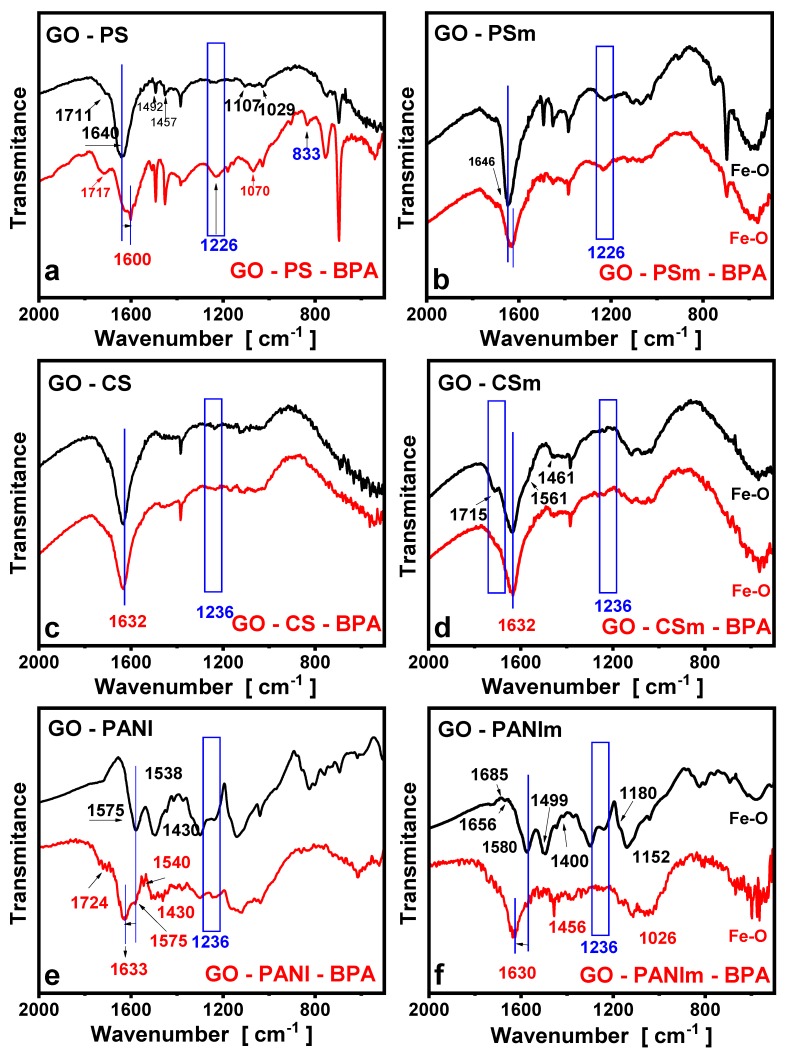
Fourier transform infrared (FTIR) spectrum for the materials before and after BPA adsorption. (**a**) GO-PS; (**b**) GO-PSm; (**c**) GO-CS; (**d**) GO-CSm; (**e**) GO-PANI; (**f**) GO-PANIm.

The FTIR spectra of the GO–PS and GO–PSm nanocomposites are presented in Figure 13a,b. The peaks at 1026 and 1667 cm^−1^ presented in the spectra, show the presence and attachment of PS chains to graphite oxide layers [58]. The peaks at 752 and 680 cm^−1^ were ascribed to the =C–H out-of-plane vibration and ring out-of-plane deformation of mono-substituted aromatic group, respectively [59]. A series of strong adsorption peaks at 1605, 1506, 1457 and 1037 cm^−1^ were all characteristic peaks of benzene in PS chains [53,55]. All these bands showed the successful impregnation of polystyrene onto the surface of GO. Additionally, for the magnetic nanocomposites, the characteristic peak presented at about 581 cm^−1^ can be attributed to the stretching vibration of the Fe – O bonds in the tetrahedral site of magnetite [6].

The FTIR spectra of GO-CS nanocomposite is presented in Figure 13c. Characteristic bands attributed to chitosan are presented at 1636 and 1561 cm^−1^ due to the stretching vibrations of the C–O of –NHCO– (amide I) as well as to the bending vibration of the N-H bond of –NH_2_, respectively [60,61]. The peaks at 1715 cm^−1^, attributed to GO carboxylic groups, were eliminated indicating the reaction of the carboxylic groups of graphite oxide with –NH_2_ groups of chitosan during the preparation of the composite [31,62]. The –NH_2_ groups of chitosan are able to react with the carboxylic and epoxidic graphite oxide’s groups [31,32]. Based on the FTIR spectra of the GO–CSm nanocomposite, it can be concluded that the carboxylic groups of GO reacted with –NH_2_ groups of magnetic chitosan to amides (1636 cm^−1^) and carboxylate groups (~1400 cm^−1^), as well as with the amino nucleophilic attack on the epoxy groups of GO, which led to the amino (1561 cm^−1^) formation. The peak at 1630 cm^−1^, characteristic of the GO sp^2^ carbon skeletal network (aromatic rings) can also indicate the formation of the Schiff bases as a result of the above described reaction [31]. In the spectra for the magnetic composite GO–CSm (Figure 13d), the characteristic peaks at 580 and 575 cm^−1^, corresponding to the Fe_3_O_4_ stretch vibrations, confirmed the successful impregnation of the magnetite [41,63].

In the spectra for GO–PANI and GO–PANIm nanocomposites (Figure 13e,f), the characteristic peak of the carboxyl groups at 1730 cm^−1^ disappeared due to the reaction of these groups with the –NH_2_ groups of the polyaniline to form amides that presented a characteristic peak at 1540 cm^−1^ [64] indicating that PANI was successfully impregnated onto GO [65,66]. The bands at 1143, 1491, and 1564 cm^−1^ may be attributed to the N−Q−N−Q stretch of the quinonoid (Q) ring, benzenoid ring vibration (C=C stretching deformations), and quinonoid ring vibration (N=Q=N), while the band at 1290 cm^−1^ is assigned to the C–N stretching vibrations correlated to emeraldine [25,29]. The characteristic peak at ~800 cm^−1^ that is related with the bending vibration of C–H group to the 1,4 substituent group benzene ring out of plane, as well as the in-plane vibration of C−H groups (987 cm^−1^), were indicative of the end-to-end connection way of polymerization of aniline, confirmed that aniline had been successfully turned into PANI [66] while the peaks due to the N−Q−N−Q stretching of the quinonoid ring were indicative that PANI had been covalently grafted onto GO surface. The magnetic counterpart besides the bands as in the spectra for the non-magnetic composite, presented the representative peak of magnetite at ~580 cm^−1^ [63].

### 3.4. Mechanism of Adsorption

No correlation was found between the specific surface area (SSA) of the prepared material and the maximum adsorption capacity of BPA. The material with the greater SSA (i.e., 35 m^2^/g for GO-PANIm) had approximately the same capacity with GO-PS, which had 4.2 times lower SSA and 0.6 times lower capacity from GO–CS, which had 38.9 times lower SSA.

Given the characteristics of BPA and of the nanocomposites prepared, the adsorption mechanisms may involve electrostatic interactions, hydrogen bonding and π–π interactions [3,67]. Additionally, the GO-polymer nanocomposites can adsorb a part of the BPA because of the hydrophobic interactions between the BPA and the adsorbents [68]. Initially, the electrostatic interactions did not affect BPA adsorption onto graphene oxide-based nanocomposites, since BPA was in its molecular form at the adsorption solution pH (3) [46]. Graphene-oxide-based nanocomposites contained hydrophobic (i.e., graphene basal planes with aromatic rings and various hydrophilic groups such as hydroxyl (–OH) and carboxyl (O=C–O) [69]. BPA also had both hydrophobic (i.e., phenyl groups) and hydrophilic (i.e., hydroxylic) functional groups [70]. Due to the comparable configuration of BPA and GO-polymer composites, it was expected that two types of adsorbent-adsorbate interactions might be responsible for the adsorption of BPA onto the composites. One probable interaction is hydrogen bonding (H-bond) between oxygen-containing groups of the materials and/or with the benzene rings on the materials surface that can also act like hydrogen bond donors and hydroxylic functional groups of BPA [70,71]. The other one may be π–π interactions between the benzene rings contained in BPA and GO-based materials. 

In order to interpret these interactions, the FTIR spectra of all nanocomposites, after BPA adsorption, are described below and compared to the above described spectra of the raw nanocomposites. In Figure 13a–f the spectra of the under-examination nanocomposites after BPA adsorption are presented along with the spectra of the raw nanocomposites. From these spectra can be seen that for the BPA loaded samples, the bands due to C–H bending in substituted aromatic rings, presented a higher intensity, indicating that BPA molecules had been laid on the nanocomposites’ surface. The peak due to the skeletal vibration of aromatic rings (i.e., C=C bonds of the benzene ring), in the spectra after BPA adsorption, presented a shift to lower wavenumbers (e.g., for the GO–PS sample there is a shifting from 1640 to 1624 cm^−1^ and for GO–PANI sample from 1656 to 1633 cm^−1^), indicating π–π interactions between π electrons of the benzene rings of BPA with the π electrons of the benzene rings of the graphene oxide [3,4,72,73]. Furthermore, new bands at about 1260 cm^−1^ appeared indicative of phenolic groups adsorbed on the nanocomposites’ surface, while the bands at 1190 cm^−1^, due to the O–H groups, shifted to lower wavenumbers, after their interaction with the OH groups of the BPA. 

It was concluded that BPA was adsorbed on the nanocomposites’ surface via the π–π interactions between the π electrons of the benzene rings of BPA and the π electrons of the benzene rings of the nanosorbents as well as hydrogen bonding between the nanocomposites and the –OH groups of BPA. Additionally, the existence of nitrogen-containing groups (i.e., amino and amines groups) in the chitosan and polyaniline composite enhances the H-bonding interactions (G-NH_2_···HO-R) [3], and thus the increase in adsorption capacity of these materials in comparison with polystyrene-based composites.

The presence of magnetite resulted in increased surface charge of the GO-PS and GO-CS magnetic nanocomposites and in decreased for GO-PANI, as was confirmed from the potentiometric titration results (Figure 7), and thus in corresponding attraction of the BPA molecules may be due to influence of the π–π attraction. This had a positive result for the adsorption efficiency of chitosan and polystyrene-based nanocomposites and a negative for the polyaniline-based material. Various mechanisms may simultaneously control BPA adsorption on graphene-oxide-based nanocomposites. In our study, the main adsorption mechanisms could be the π–π interactions while the hydrophobic interactions and H-bonding could enhance the BPA adsorption. The same mechanisms for BPA adsorption were suggested by other authors for materials with chitosan [74,75] and polystyrene [76] and polyaniline [26].

## 4. Conclusions

The adsorption of bisphenol A, a commonly industry-used EDC, from aqueous solutions onto graphene-oxide-based magnetic nanocomposites with polymers was investigated. The adsorption isotherms were evaluated by the Langmuir and Freundlich models with the Langmuir isotherm to fit better, indicative of a monolayer adsorption. The maximum adsorption capacities, at 25 °C, were found to be 36.27 mg/g for GO–PS, 50.25 mg/g for GO–PSm, 54.18 mg/g for GO–CS, 86.22 mg/g for GO–CSm, 51.56 mg/g for GO–PANI, 31.76 mg/g for GO–PANIm. The adsorption of Bisphenol A onto GO-based materials, depended more on the initial BPA concentration, contact time and less to the solution pH. The textural characteristics of the materials were not important parameters for the adsorption of BPA. The adsorption was found to be dependent by the oxygen-containing functional groups of graphene nanosheets as well as the amino groups of the polymers used. Interaction like π–π interactions and H-bonding were involved in the adsorption. The graphene oxide-based magnetic nanocomposites presented sufficient adsorption capacities for BPA. 

## Figures and Tables

**Table 1:** Textural parameters for the graphite oxide (GO)-polymers composites.

Adsorbent	SSA m^2^/g	V_tot_ (cm^3^/g)	V_mic_ (cm^3^/g)	V_mes_ (cm^3^/g)
**GO**	20.93	0.088	0.064	0.024
**GO–PS**	8.35	0.061	0	0.061
**GO–PSm**	10.00	0.059	0	0.059
**GO–CS**	0.89	0.099	0	0.099
**GO–CSm**	5.32	0.034	0	0.034
**GO–PANI**	27.02	0.260	0	0.260
**GO–PANIm**	35.78	0.300	0	0.300

**Table 2:** Kinetic parameters for the adsorption of bisphenol–A (BPA) onto GO-polymer composites.

Adsorbent	Pseudo-First Order			Pseudo-Second Order		
	k_1_ (min^−1^)	q_e_ (mg/g)	R^2^	k_2_ (min^−1^)	q_e_ (mg/g)	R^2^
**GO–PS**	0.0991	19.20	0.947	0.0083	20.49	0.997
**GO–PSm**	0.1951	32.95	0.936	0.0083	36.04	0.976
**GO–CS**	0.0486	46.28	0.968	0.0011	52.66	0.992
**GO–CSm**	0.0320	69.12	0.934	0.0004	81.26	0.976
**GO–PANI**	0.1260	44.91	0.948	0.0039	48.49	0.980
**GO–PANIm**	0.0161	25.15	0.912	0.0003	34.71	0.895

**Table 3:** Equilibrium parameters for the adsorption of BPA on GO-polymer composites.

Adsorbent	Langmuir Model			Freudlich Model		
	Q_max_ (mg/g)	K_L_	R^2^	K_F_	1/n	R^2^
**GO**	17.27	0.172	0.934	5.03	0.299	0.772
**GO–PS**	36.27	0.044	0.972	5.24	0.374	0.853
**GO–PSm**	50.25	0.020	0.990	3.82	0.468	0.942
**GO–CS**	54.18	0.053	0.997	12.07	0.291	0.952
**GO–CSm**	86.22	0.037	0.989	9.70	0.436	0.931
**GO–PANI**	51.56	0.045	0.995	8.81	0.340	0.944
**GO–PANIm**	31.76	0.050	0.991	6.49	0.303	0.961

**Table 4:** Comparison of BPA adsorption on Mag-PVP with other adsorbents.

Adsorbent	Q_max_ (mg/g)	pH	Reference
Magnetite (Fe_3_O_4_)	5.1	2.8–8.5	[49]
GO	17.3	3	[3]
Magnetic chitosan fly-ash-cenospheres	31.9	7	[50]
Porous carbon produced at 1000 °C from Moso bamboo	41.8	–	[51]
Magnetic carbon nanotubes	45.3	6.2	[49]
Modified carbon nanotubes	70	6	[52]
Reduced GO (RGO)	80.8	3	[3]
Graphene	94.1	3	[3]
Magnetic–polydivinylbenzene	115.9	6–7	[53]
Potato peels derived activated carbon (KOH activation)	190.7	3	[4]
MAP–GBM	324.0	7	[54]
Potato peels derived activated carbon (H_3_PO_4_ activation)	445.9	3	[4]
GO–CSm	86.2	3	This study
GO–PSm	50.25	3	This study
GO–PANIm	31.8	3	This study
